# Adaptive Prediction Error Coding in the Human Midbrain and Striatum Facilitates Behavioral Adaptation and Learning Efficiency

**DOI:** 10.1016/j.neuron.2016.04.019

**Published:** 2016-06-01

**Authors:** Kelly M.J. Diederen, Tom Spencer, Martin D. Vestergaard, Paul C. Fletcher, Wolfram Schultz

**Affiliations:** 1 Department of Physiology, Development, and Neuroscience, University of Cambridge, Downing place, Cambridge CB2 3DY, United Kingdom; 2 Department of Psychiatry, University of Cambridge, Robinson Way, Cambridge CB2 0SZ, United Kingdom; 3 Cambridgeshire and Peterborough NHS Foundation Trust, Cambridge CB21 5EF, United Kingdom

**Keywords:** reward, standard deviation, normalization, performance, fMRI

## Abstract

Effective error-driven learning benefits from scaling of prediction errors to reward variability. Such behavioral adaptation may be facilitated by neurons coding prediction errors relative to the standard deviation (SD) of reward distributions. To investigate this hypothesis, we required participants to predict the magnitude of upcoming reward drawn from distributions with different SDs. After each prediction, participants received a reward, yielding trial-by-trial prediction errors. In line with the notion of adaptive coding, BOLD response slopes in the Substantia Nigra/Ventral Tegmental Area (SN/VTA) and ventral striatum were steeper for prediction errors occurring in distributions with smaller SDs. SN/VTA adaptation was not instantaneous but developed across trials. Adaptive prediction error coding was paralleled by behavioral adaptation, as reflected by SD-dependent changes in learning rate. Crucially, increased SN/VTA and ventral striatal adaptation was related to improved task performance. These results suggest that adaptive coding facilitates behavioral adaptation and supports efficient learning.

## Highlights

• Midbrain and Ventral Striatum code prediction errors relative to reward variability
• Adaptive prediction error coding in the midbrain developed across trials
• Adaptive coding predicts behavioral adaptation to reward variability
• Increased adaptation is associated with improved task performance

## Teaser

Diederen et al. show that the Substantia Nigra/Ventral Tegmental Area complex and Ventral Striatum code prediction errors relative to reward variability during learning. Improvements in such adaptation are associated with superior task performance, suggesting that adaptive coding benefits learning.

## Introduction

Learning to accurately predict upcoming reward is essential for decision making. A critical challenge during learning is that most reward fluctuate from one moment to the next (i.e., reward are elements of probability distributions with a certain mean and SD) (Schultz et al., 2008). Consequently, prediction errors not only indicate the extent to which our predictions are wrong, but also represent the extent of fluctuation in reward value. Since it would be sub-optimal to update predictions too readily when the prediction error signal itself is unreliable, effective error-driven learning requires individuals to adapt to reward variability. Such adaptation may be accomplished through the use of SD-dependent learning rates or via the direct scaling of prediction errors (Diederen and Schultz, 2015).

The wealth of studies reporting prediction error coding in midbrain dopaminergic nuclei and the ventral striatum render it conceivable that prediction errors are directly scaled by SD. Scaled prediction error coding optimally exploits the limited coding capacity of the brain by tuning it to the expected variability of these errors (Tobler et al., 2005). By tuning coding capacity relative to the SD of the predicted distribution, the gain (i.e., the relationship between prediction error size and neural responses) adapts, and neural sensitivity is optimized for detection of smaller differences when the variability of possible prediction errors is smaller (Kobayashi et al., 2010). Indeed, prediction error responses in monkey midbrain dopamine neurons do not code the simple difference between reward and prediction but adapt to the probability distribution of predicted reward (Tobler et al., 2005). In addition, when reward contingencies are made explicit, BOLD responses in the human striatum vary with the probability (high versus low) and sign (positive versus negative) of prediction errors independently of prediction error magnitude (Bunzeck et al., 2010, Park et al., 2012). Although these studies provide preliminary support for adaptive prediction error coding, it is critical to investigate adaptive coding during learning, as adaptation should serve to make learning more efficient. In addition, it is unknown whether prediction error responses in the human brain adapt to the SD of these errors and whether such adaptation benefits learning.

**Figure 1:**
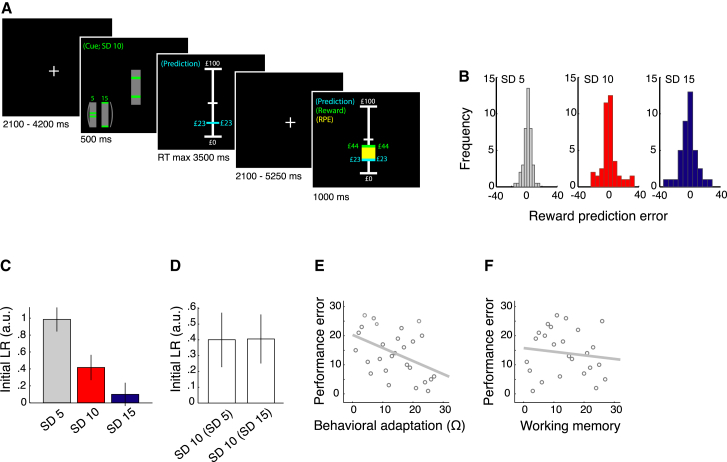
Experimental Task and Behavioral Results (A) Participants predicted the magnitude of upcoming reward as closely as possible from the past reward history. Vertical bar cues signaled whether rewards would be drawn from a distribution with small, medium, or large variability. After stating their prediction, participants received a reward, displayed in green. A yellow bar spanning the distance between the predicted and the received reward indicated the reward prediction error (RPE). (B) Experienced RPEs averaged across all participants. An increase in the fluctuation of reward value was associated with an increase in the range and SD of experienced RPEs indicating that the experimental manipulation was successful. (C) Initial learning rates for the Pearce-Hall model decreased significantly for increases in SD, suggesting behavioral adaptation to reward variability. Data were *Z* scored per participant across SDs to control for potential outliers. Thus, initial learning rate data are presented in a.u. Bar graphs depict average ± SEM initial learning rates. (D) Initial learning rates for SD 10 conditions did not depend on the magnitude of the second SD within a session (i.e., SD 5 or SD 15), suggesting an absence of contextual effects on initial learning rates. Data were *Z* scored per participant across the two SD 10 conditions to control for potential outliers. Bar graphs depict average ± SEM initial learning rates. (E) Increased behavioral adaptation correlates with decreased performance error, indicating improved performance with adaptation. To quantify behavioral adaptation (ranked), we determined whether SD^−1^ was a significant predictor of learning rates: *β*_0_ + *β*_1_ SD^−1^. The higher is R^2^ the better is SD a predictor of learning rate. (F) Performance error did not depend on working memory capacity measured using the Wechsler reverse Digit Span task. RPE, reward prediction error; RT, reaction time; LR, learning rate. Ω, behavioral adaptation.

Here, we investigated whether prediction error responses can adapt to reward variability during learning in the human midbrain (substantia nigra/ventral tegmental area [SN/VTA] complex) and ventral striatum, areas implicated in reward prediction error (RPE) coding, and whether efficient adaptation benefits learning. We also addressed the alternative hypothesis that behavioral adaptation is facilitated by SD-dependent learning rate coding. The experimental design was modified from a recent study that showed behavioral adaptation to reward variability in humans (Diederen and Schultz, 2015). During fMRI data acquisition, participants explicitly predicted the expected magnitude of upcoming rewards that were drawn from distributions with different SDs (i.e., 5, 10, or 15). We used explicit symbolic cues to indicate the relative magnitude of reward variability (i.e., small, medium, large); however, participants were unaware of the exact SDs, which thus had to be learned. Each SD was paired with two different means resulting in a total of six conditions. In each of three task sessions, participants alternatingly predicted reward from one of two conditions, each with a different SD. After each prediction participants received a reward (see Figure 1A for an example trial). The explicit presentations of prediction and reward enabled us to compute and display the RPE on each trial. Trial-by-trial variation in RPE magnitude ensured that the prediction errors covered the whole range of potential errors (Figure 1B).

BOLD responses in the human midbrain (SN/VTA) and ventral striatum adapted to the variability of prediction errors, as reflected in steeper prediction error coding slopes when the SD was lower. Subsequent analyses suggested that prediction errors were encoded as a function of SD as BOLD responses varied with normalized rather than absolute RPEs. We found no support for the alternative hypothesis that the adaptive process is mediated through coding of SD-dependent learning rates. SN/VTA adaptive prediction error coding was not immediate but emerged as trials progressed. Importantly, the individual degree of adaptive coding in the SN/VTA complex and ventral striatum correlated with behavioral measures of adaptation and was predictive of performance.

## Results

### Behavior

Participants indicated the expected magnitude of upcoming reward on every trial of the task. Following reward prediction, the computer revealed the actual reward that was drawn from an approximate Gaussian distribution. Thus, on every trial the participants experienced a prediction error (reward received–reward predicted). Optimal updating of reward predictions would require participants to infer the expected value (EV) of the reward distributions using Bayesian mean-tracking. Thus, Bayesian mean-tracking constituted our null model. In this model, rewards are assumed to be drawn from distributions with variance *σ*^2^, which was a free parameter that was estimated separately for each SD condition (see Supplemental Experimental Procedures; Table S1). Given that Bayesian mean-tracking is computationally demanding, a biologically plausible alternative mechanism for updating predictions is reinforcement learning. Formal model comparisons revealed that participants were more likely to use reinforcement learning compared to Bayesian mean-tracking (see Table 1 for model comparisons using Akaike and Bayesian information criteria [AIC/BIC]). Specifically, participants’ prediction sequences were best predicted by a dynamic learning rate Pearce-Hall (PH) reinforcement learning model (Table 1). The PH learning rate depends on the weighted, unsigned, prediction error across the past trials and a decay constant. Thus, earlier observations are considered more informative than later observations. Based on the superior fit of this model, we used parameters estimated for this model in subsequent analyses.

### SD Impacts on Learning Behavior

Fitted learning rates decreased as SD increased (F (2, 52) = 6.54, p = 0.003) (Figure 1C), an effect that was most pronounced for the smallest SDs (i.e., SD 5 versus SD 10; T (26) = 2.20, p = 0.018; SD 10 versus SD 15; T(26) = 1.27, p = 0.108). These results suggest a non-linear effect of SD on learning rate. Learning rates for SD 10 conditions did not depend on the SD of the other condition within a session (SD 5 or SD 15; T(26) = 0.023, p = 0.509) (Figure 1D), which argues against contextual effects on learning rates. In addition, the decay in learning rate did not vary across SD conditions, suggesting that SD-dependent differences in learning rate did not change as trials progressed (p > 0.1; Figure S2). To formally test for behavioral adaptation, we extended the PH model by including a scaling parameter on prediction errors (Diederen and Schultz, 2015). Model comparisons using AIC and BIC showed that this adaptive PH model outperformed all other models; the non-adaptive PH model was the second best model (Table 1). The comparison using BIC only provided marginal evidence in favor of the adaptive PH model; we therefore conducted a fixed effects likelihood ratio test, as previously reported (Li et al., 2011), to examine the extent to which the difference in model fit between the two PH variants was significant. This test revealed that the adaptive PH model significantly outperformed the non-adaptive PH model ($\chi_{27}^{2}$ = 156.73). Inspection of estimated scaling parameters showed that these parameters differed significantly from zero and that participants scaled prediction errors relative to, but with a smaller magnitude than log(SD) (T(31) = 8.876, p < 0.001) (Figure S2). Such behavioral adaptation to SD makes it likely that prediction errors are encoded relative to SD, and we have suggested that it facilitates efficient learning (Diederen and Schultz, 2015). Indeed, participants who showed decreased learning rates with increased SD presented with lower performance errors (|prediction − EV|) across all trials (Spearman’s ρ = −0.455, p = 0.009) (Figure 1E). Individual differences in performance did not result from variations in individual working memory capacity as measured using the Wechsler reverse Digit Span task (Wechsler, 1958) (Spearman’s ρ = −0.123, p = 0.270) (Figure 1F). These results confirm that prediction errors scale to reward variability and that such adaptation benefits learning.

### Adaptive Coding

**Figure 2:**
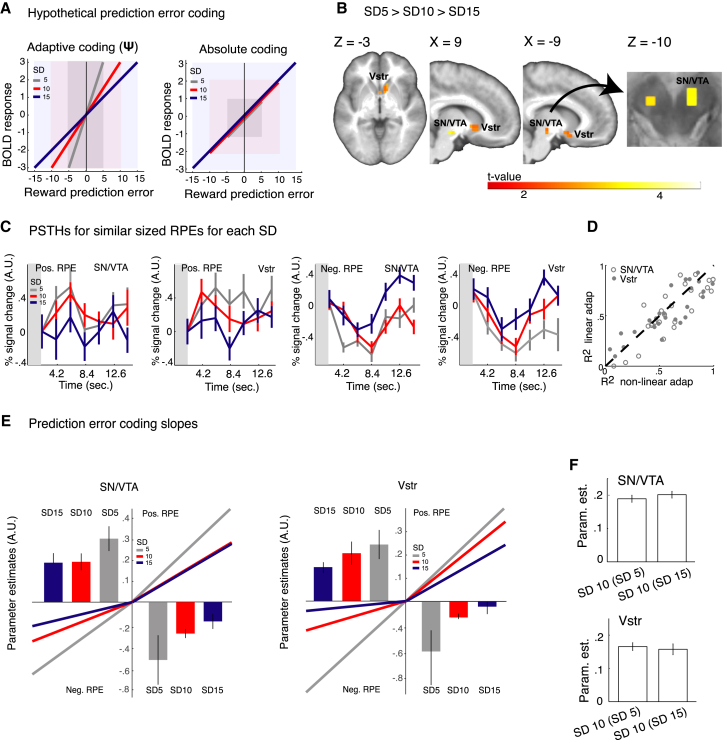
Adaptive Prediction Error Coding (A) Schematic of adaptive coding versus absolute coding of RPEs. Left: Hypothesized slopes for adaptive coding of RPEs. If the brains limited coding capacity is tuned to a larger range of RPEs, BOLD responses should increase less with a certain increase in RPE. Thus, the brains’ sensitivity to detect small changes in RPEs would be reduced in distributions with a larger SD. Right: Hypothesized slopes for absolute coding of RPEs. In the absence of adaptive coding, RPE slopes should be similar for the different SDs. (B) Adaptive RPE coding: small > large SDs (i.e., SD^−1^ centered at zero). RPE slopes increased when SD decreased, in line with adaptive coding of RPEs. Significant effects were observed in the midbrain (SN/VTA) complex and ventral striatum ROI (p < 0.05 FWE small volume correction [SVC]). For visual presentation only, we lowered the threshold to p = 0.1 FWE. (C) Increased average (± SEM) responses (peristimulus time histograms [PSTHSs]) to similar sized (positive and negative; ± 12 and −12) RPEs in SD conditions with a lower SD, in line with adaptive coding. To obtain these time courses, we binned trials associated with RPEs between 5 and 15 and between −5 and −15 for each condition and participant. Subsequently, we extracted PSTHs at individual peak voxels displaying adaptive coding for positive and negative RPEs. (D) A non-linear adaptive model (SD^−1^) provided a superior fit of RPE slopes compared to a linear adaptive model, in line with the non-linear decrease in initial learning rate for increases in SD. (E) RPE coding slopes. Increase in average (± SEM) RPE coding slopes and median % signal change when SD is smaller. We displayed both the average and median for completeness. Coding slopes for the midbrain (SN/VTA) and ventral striatum were averaged over all voxels in the a-priori-defined ROIs. Data were *Z* scored per participant across SDs to control for potential outliers. (F) Average (± SEM) RPE coding slopes for SD 10 conditions did not depend on the SD of the second condition in a session (SD 5 or SD 15), suggesting that there were no contextual effects on RPE coding. Data were *Z* scored per participant across SD 10 conditions to control for potential outliers. Vstr, ventral striatum; SN/VTA, substantia nigra/ventral tegmental area; param. est., parameter estimates; ROI, region of interest; Ψ, neural adaptation. Although we used different tests to establish neural adaptation, we used Ψ to refer to neural adaptation independently of the specific test used.

If the brain’s limited coding capacity is relieved by tuning to more variable prediction errors (i.e., adaptive coding), this should result in smaller neural prediction error coding slopes for larger SDs (Figure 2A left). In the absence of adaptive coding, regression slopes would be similar for the different SDs (Figure 2A, right). The non-linear relationship between SD and initial learning rates suggests a similar non-linear decrease in prediction error slopes across SDs. Using a contrast that reflected such non-linearity (i.e., 1/SD, centered at zero), we observed that SN/VTA activity increased more with increases in prediction error magnitude in SD 5 conditions compared to SD 10 and SD 15 conditions (Main effect SD: −8, −18, −10, Z = 3.46/ 3.40 for the 8 mm and 6 mm smoothing, respectively, p < 0.05 FWE, small volume correction [SVC]) (Figures 2B–2E). A similar effect was observed in the ventral striatum (−18, 1, −10, Z = 3.54/ 3.54 for the 8 mm 6 mm smoothing, respectively, p < 0.05 FWE SVC) (Figures 2B and 2C). A linear adaptive contrast (i.e., 1, 0, −1) on prediction error regression slopes revealed a similar but somewhat less significant result compared to the non-linear contrast (SN/VTA: max. Z: 2.87/2.86 for the 8 mm and 6 mm smoothing, respectively; ventral striatum: 3.17/3.14 for the 8 mm and the 6 mm, respectively). We observed no significant effect of SD on prediction error coding slopes in the cerebellar *control* ROI that had the same dimensions as the experimental ROI (all p > 0.1 SVC), suggesting that adaptation in the a-priori-defined ROI did not merely result from the more liberal multiple comparisons correction. Whole-brain analyses (p < 0.05 cluster level) revealed additional activation for the main effect of SD in a cluster comprising the parahippocampal gyrus, the lentiform nucleus, and the thalamus and a second cluster that included the left superior temporal gyrus and the ventrolateral and dorsolateral prefrontal cortices (Table 2). ROI analyses (averaged over all voxels in the a-priori-defined ROIs) confirmed that SD-specific prediction error slopes decreased non-linearly with increases in SD (comparison of R^2^ for a linear [1, 0, −1] and non-linear model [1/SD centered at zero]: T(53) = 2.2340, p = 0.0149) (Figure 2D). In line with the behavioral results, prediction error coding slopes for SD 10 conditions did not depend on the SD of the second condition within a session (SD 5 or SD 15; all p > 0.1 FWE SVC, cluster and voxel-wise analyses; ROI analysis: T(53) = 0.8763, p = 0.1924) (Figure 2F). These results suggest that prediction error coding slopes adapt to SD and not to the context.

### Prediction Errors Are Encoded Relative to SD

**Figure 3:**
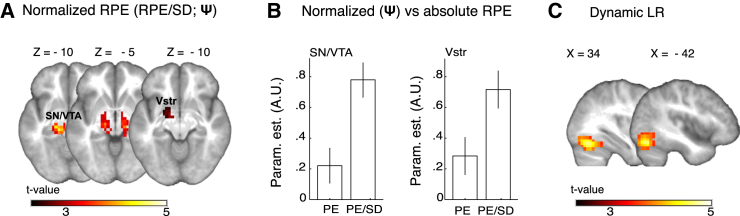
Prediction Errors Are Coded Relative to SD (A) Normalized RPE responses. Significant coding of normalized RPEs (I.e., RPE/SD) after removing all shared variance between normalized and non-normalized RPEs. To facilitate comparison of normalized and non-normalized regressors, parametric modulators were *Z* scored prior to model estimation. *Z* scores were calculated per subject, across all SD conditions. Whereas BOLD responses were significant on cluster level as well as in the a-priori-defined ROI in the midbrain (SN/VTA complex), activity in the ventral striatum only became significant when we decreased the search volume to a 9-mm sphere centered on the peak area showing significant adaptation in the previous analysis (see Figure 2). (B) Increased average (± SEM) RPE coding slopes (parameter estimates) for normalized RPEs compared to (non-normalized) RPEs in the SN/VTA complex and ventral striatum. Coding slopes for the SN/VTA complex and ventral striatum were averaged over all voxels in the a-priori-defined ROI. Data were *Z* scored per participant across parameter estimates for normalized and non-normalized RPEs to control for potential outliers. (C) Significant learning rate coding slopes in a cluster including the occipital cortex and cerebellum. Vstr, ventral striatum; SN/VTA, substantia nigra/ventral tegmental area; param. est., parameter estimates. Ψ, neural adaptation.

The decrease in prediction error coding slopes for larger SDs suggests that prediction errors are encoded relative to the SD of reward. Thus, we sought to establish whether a normalized code (prediction error/SD) would be superior in explaining variations in BOLD responses compared to a non-normalized code. After removing all variance shared by normalized and non-normalized prediction errors, BOLD responses no longer varied significantly with non-normalized prediction errors neither in the SN/VTA and ventral striatum (p > 0.9 FWE SVC), nor at whole-brain level (p > 0.1 FWE cluster and voxel-wise correction). In sharp contrast, normalized prediction errors still tracked BOLD responses in a midbrain cluster that included the SN/VTA complex and extended into the hypothalamus (−14, −26, −10, Z = 4.35, p < 0.05 FWE cluster correction and −11, −18, −6, Z = 3.07/3.06 for the 8 mm and 6 mm smoothing, respectively, p < 0.05 FWE SVC) (Figure 3A). The ventral striatum did not significantly code normalized prediction errors when all shared variance between non-normalized and normalized prediction errors was removed (−14, 8, −14, Z = 2.50/2.48, p = 0.190/0.196 FWE SVC for the 8 mm and 6 mm smoothing, respectively). However, when we restricted the search volume to the cluster showing significant adaptive coding in the previous, less conservative analysis by drawing a 9 mm sphere centered on the coordinate of maximum activation in that analysis, we observed significant normalized prediction error coding (−14, −8, −6, Z = 2.94/ 2.92 for the 8 mm and 6 mm smoothing, respectively, p < 0.05 FWE) (Figure 3A). ROI analyses revealed a significant increase in coding slopes for normalized compared to non-normalized prediction errors in the a-priori-defined SN/VTA complex (Wilcoxon signed rank = 431, Z = 2.682, p = 0.007) and ventral striatal ROI (Wilcoxon signed rank = 416, Z = 2.811, p = 0.005) (Figure 3B). We observed no significant effects in the control ROI (p > 0.1), thus suggesting that adaptation in the a-priori-defined ROI did not merely result from the more liberal multiple comparisons correction. These results suggest that prediction errors are coded relative to reward variability in the human SN/VTA and to a lesser extent in the ventral striatum.

### Learning Rate Coding

It has to be noted that although a superior fit of the adaptive PH model indicated scaling of prediction errors relative to SD, computational modeling cannot distinguish between prediction error scaling and learning rate scaling. Thus, the observed behavioral adaptation may be facilitated by SD-dependent learning-rate coding rather than normalized prediction error coding. BOLD responses in two large clusters encompassing the bilateral cerebellum and inferior occipital gyrus varied significantly with trial-by-trial PH learning rates (34, −68, −18, Z = 4.22; −42, 72, −18, Z = 4.17, p < 0.05 FWE cluster-level correction) (Figure 3C). This effect did not depend on the SD of reward distributions in either the a-priori-defined ROI or at whole-brain level (all p values > 0.1), arguing against scaled learning-rate coding underlying behavioral adaptation to reward variability. Thus, these results suggest that the effect of SD on learning is incorporated via the scaling of prediction errors, not learning rate. In addition, as the PH learning rate decays in a trial-wise manner, these results suggest that the effect of trial number on learning is facilitated via the coding of dynamic learning rates. Indeed, a parametric modulator that scaled prediction errors relative to both SD and the trial-wise decay in learning rate did not provide a better fit of the fMRI data compared to a parametric modulator that only normalized prediction errors to reward variability in either the a-priori-defined ROI or at whole-brain level (p > 0.1). In addition, prediction errors that were scaled by dynamic learning rates (but not SD) did not provide a better fit of the fMRI data compared to unscaled prediction errors. These results confirm that the effect of trial number is regulated via the learning rate, whereas the effect of SD is incorporated through the use of scaled prediction errors.

### Timescale Adaptive Coding

**Figure 4:**
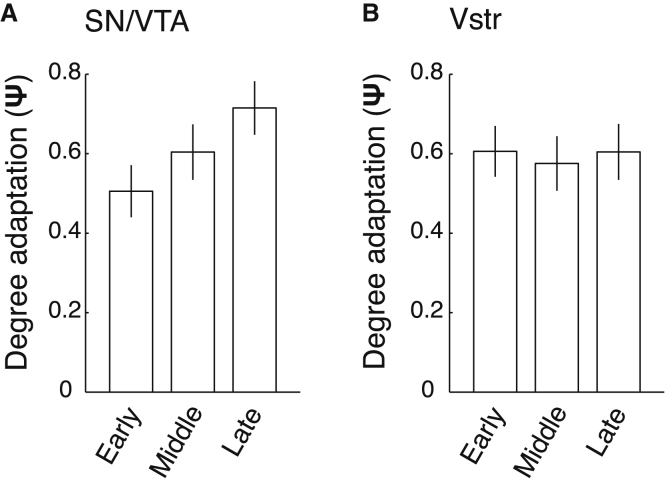
Adaptive Coding Emerges across Trials in the SN/VTA (A) Average (± SEM) adaptive coding in the midbrain (SN/VTA) ROI increased for later compared to earlier trials. (B) Average (± SEM) adaptive coding in the ventral striatal ROI did not vary with later compared to earlier trials. SN/VTA, substantia nigra/ventral tegmental area; Vstr, ventral striatum; Ω, behavioral adaptation; Ψ, neural adaptation. Early, Middle and Late, Early trials, Middle trials, Late trials.

As the adaptive process conceivably requires time, we investigated adaptive coding to SD during early, middle, and late trials. Although the non-linear adaptive model provided a good description of SD-specific prediction error coding slopes for each of the different task phases, adaptive coding increased for late compared to early trials in the SN/VTA a-priori-defined ROI (F(1,26) = 6.85, p = 0.015) (Figure 4A). In strong contrast, adaptive coding was highly similar for early and late trials in the ventral striatal ROI (F(1,26) = 0, p = 0.989) (Figure 4B). These results show a clear distinction between adaptive coding in the SN/VTA complex and ventral striatum and render it likely that adaptation of RPEs in the SN/VTA complex does not occur instantaneously.

### Behavioral Adaptation, Adaptive Coding, and Performance

**Figure 5:**
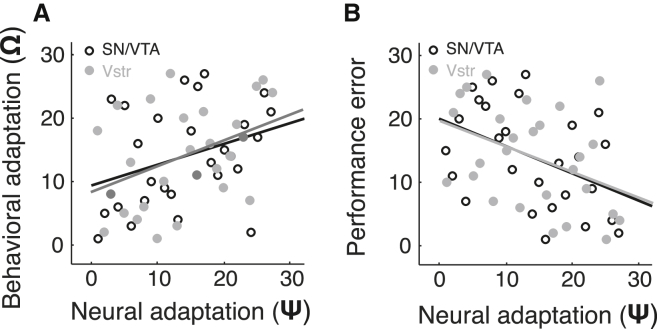
Neural Adaptation Correlates with Behavioral Adaptation and Performance (A) Superior behavioral adaptation to reward variability is associated with improved neural adaptation in the SN/VTA complex and ventral striatum. To quantify behavioral and neural adaptation, we determined whether SD^−1^ was a significant predictor of learning rates and RPE slopes: *β*_0_ + *β*_1_ SD^−1^. The higher R^2^ is, the better SD serves as a predictor of learning rate and RPEs. (B) Superior neural adaptation in the SN/VTA complex and ventral striatum correlates with decreases in performance error (|prediction − EV| averaged across all SDs and trials). SN/VTA, substantia nigra/ventral tegmental area; Vstr.; ventral striatum. Ψ, neural adaptation.

The observed behavioral scaling of prediction errors to reward variability may be facilitated by adaptive coding to the SD of RPEs. Indeed, the degree of behavioral adaptation varied significantly with individual differences in adaptive coding in the SN/VTA complex (Spearman’s ρ = 0.329, p = 0.047) (Figure 5A) and ventral striatum (Spearman’s ρ = 0.406, p = 0.018) (Figure 5A).

Importantly, adaptive coding should not only facilitate behavioral adaptation to reward variability but should also serve to make learning more efficient. Thus, we investigated whether the individual degree of adaptive prediction error coding was related to task performance. Participants displaying a higher degree of adaptive prediction error coding in the SN/VTA complex and the ventral striatum outperformed participants with a lower degree of adaptation (Spearman’s ρ = −0.431, p = 0.013; Spearman’s ρ = −0.407, p = 0.018 for the SN/VTA and ventral striatum, respectively) (Figure 5B). The tight relationship between behavioral adaptation and adaptive coding suggests that adaptive coding of prediction errors underlies behavioral adaptation and facilitates learning.

### Positive and Negative Prediction Errors

**Figure 6:**
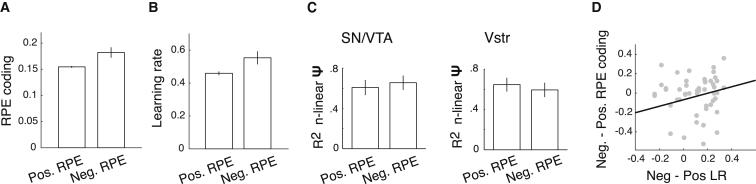
Adaptive Prediction Error Coding Is Consistent across Positive and Negative Prediction Errors (A) Average (± SEM) RPE slope magnitude was significantly higher for negative compared to positive RPEs (results collapsed over the SN/VTA complex and ventral striatum). (B) Average (± SEM) estimated learning rates were significantly higher for negative compared to positive RPEs. (C) The average (± SEM) degree of neural adaptation did no differ significantly between positive and negative RPEs in the SN/VTA complex and ventral striatum. (D) Significant positive correlation between the effect of RPE error sign on learning rates and its effect on RPE coding slopes. n-linear, non-linear; SN/VTA, substantia nigra/ventral tegmental area; Vstr, ventral striatum; LR, learning rate; Ψ, neural adaptation.

As previous work indicated differences in the coding of positive versus negative prediction errors (D’Ardenne et al., 2008), we inspected the effect of prediction error sign on BOLD responses. Prediction error coding slopes varied more with negative compared to positive prediction errors after accounting for the effect of SD (F(1,320) = 4.60, p = 0.033) (Figure 6A). However, the effect of SD on prediction error coding slopes (i.e., adaptive coding) did not depend on the sign of the prediction error (T(53) = 0.045, p = 0.964) (Figure 6B), which suggests that adaptation was consistent across positive and negative prediction errors. To investigate whether participants’ behavior varied with the sign of prediction errors, we fitted a simple Rescorla-Wagner (RW) reinforcement-learning model with separate learning rates for positive and negative prediction errors to participants’ prediction sequences. Learning rates were significantly higher for negative compared to positive prediction errors after accounting for the effect of SD (F(1,158) = 5.47, p = 0.021) (Figure 6C).

To characterize the relationship between behavioral and fMRI markers of prediction error sign, we measured correlations between individual learning rates for positive compared to negative prediction errors and differences in adaptive coding for positive versus negative prediction errors. We observed a significant positive relationship between the effect of prediction error sign on learning rates and its effect on prediction error coding slopes (Pearson’s r = 0.260, p = 0.029; in the a-priori-defined ROI that comprised the SN/VTA and ventral striatal ROI) (Figure 6D). These results indicate that individuals who weighted negative prediction errors more heavily during learning also showed stronger neural coding of negative prediction errors compared to positive prediction errors.

## Discussion

We investigated adaptation of BOLD responses to the SD of prediction errors during learning. Our data show that BOLD responses in the human midbrain (SN/VTA complex) and ventral striatum can adapt to the SD of prediction errors. In the SN/VTA, this effect only emerged as the task progressed. Thus, the magnitude of BOLD responses to a given prediction error became smaller when prediction errors fluctuated with a larger SD. Importantly, individual variability in this sensitivity was observed, and those individuals showing stronger adaptive coding in the SN/VTA and ventral striatum also showed improved behavioral adaptation, and they were able to make more accurate predictions.

The tight relationship between adaptive prediction error coding and task performance supports the hypothesis that adaptive coding serves to make learning more efficient. Weighting prediction errors with SD is critical as the size of the prediction error is meaningless without an estimate of its precision. Specifically, a prediction error of a certain size is less informative in situations where rewards fluctuate more (Diederen and Schultz, 2015). Thus, efficient learners should code prediction errors relative to SD. Such adaptive coding supports the entire dynamical range of neural systems and ensures similar BOLD responses to the highest and lowest prediction error in each distribution independently of the absolute magnitudes. As such, BOLD responses should increase similarly for increases in normalized prediction error across conditions but increase less for a certain absolute increase in prediction error when the SD is larger. This process facilitates optimal sensitivity to detect expected differences in prediction errors for each SD and makes optimal use of neurons’ limited dynamic firing range. Indeed, we found that participants represented prediction errors adaptively by differential prediction error coding slopes between the different reward distributions: steeper coding slopes for narrower distributions. After normalizing prediction errors to SD, prediction error coding slopes were similar across SD conditions, confirming adaptation to SD. Moreover, the finding of a high correlation between individual adaptive coding and behavioral adaptation suggests that adaptive coding facilitates behavioral adjustment to reward variability.

Although earlier studies did not investigate adaptive coding during learning, our results are partly in line with a previous study that showed that striatal BOLD responses varied with the probability (high versus low) of reward, but not with prediction error magnitude (Park et al., 2012). In addition, previous studies showed adaptive coding of reward value in the striatum, middle temporal gyrus, medial prefrontal cortex, orbitofrontal cortex, inferior parietal lobule, and posterior cingulate (Bunzeck et al., 2010, Cox and Kable, 2014, Elliott et al., 2008, Nieuwenhuis et al., 2005). Even though these studies focused on reward value, reward value and prediction error magnitude are typically correlated. As these studies did not separate value from prediction errors, these results could reflect prediction error coding rather than reward value adaptation. Strikingly, none of these studies reported adaptive coding in the human SN/VTA, which is critically involved in prediction error coding. The fact that reward contingencies were explicit in these studies and did not have to be learned might explain this divergence in findings. As adaptive coding is essential for learning, it may be more prominent during the learning process. Moreover, in the current study, reward distributions alternated in short blocks of four to six trials rather than trial-wise alteration as used in previous fMRI studies. This difference may be crucial, as a previous study in non-human primates showed that adaptation increased with longer task blocks (Kobayashi et al., 2010), suggesting that repetition of stimulus conditions is required to reveal adaptive coding. In addition, whereas the current study investigated BOLD responses across the whole range of potential errors, most studies solely investigated binary coding (high versus low) of reward value and prediction errors. It is unlikely that our finding of adaptive coding in the SN/VTA is spurious as an electrophysiology study in non-human primates showed that midbrain dopamine neurons adapt to the probability of predicted reward (Tobler et al., 2005). These findings add to previous studies that described adaptive coding across a wide range of species and sensory processes, thus suggesting that adaptive coding constitutes a general process for facilitating efficient coding (Carandini and Heeger, 2012).

The finding that adaptive coding emerged across subsequent task blocks in the SN/VTA converges with an earlier study in non-human primates (Kobayashi et al., 2010). Here, the fraction of neurons adaptively coding reward in the orbitofrontal cortex increased with the number of subsequent trials per task block (Kobayashi et al., 2010). Indeed, SN/VTA adaptation became most apparent during later trials in the current study. However, the adaptive model already provided a good fit of prediction error slopes during early trials, a finding that was paralleled by differences in initial learning rates across SD conditions. It is likely that this early adaptation arose from the use of explicit SD cues and generalization of learning from the practice sessions. Interestingly, adaptation did not increase across trials in the ventral striatum. A number of studies have shown divergent responses of the SN/VTA and the ventral striatum in reward tasks (D’Ardenne et al., 2008, Klein-Flügge et al., 2011, O’Doherty et al., 2006), findings that have been taken to suggest that striatal prediction error and reward value representations may not be mediated exclusively by an afferent dopaminergic signal (O’Doherty et al., 2006). Indeed, the ventral striatum receives input from areas other than midbrain dopaminergic neurons including the amygdala, orbital prefrontal cortex, insular cortex, and cingulate cortex (Haber, 2011). Moreover, it is possible that activity observed in the SN/VTA does not directly reflect the activity of intrinsic dopamine neurons but rather reflects activity within inputs to this area (Logothetis et al., 2001, O’Doherty et al., 2006). We did not observe increases in behavioral adaptation as trials progressed. This difference between behavioral and neural adaptation may reflect increased sensitivity of fMRI compared to behavioral analyses (Wilkinson and Halligan, 2004).

The inclusion of two conditions with a different SD in each session allowed us to investigate the effect of context on learning rates and prediction error slopes. Rather than coding prediction errors relative to SD, learning rates and prediction error coding could adapt to the relative SD (i.e., lowest/ highest) within a session. Specifically, SD 10 conditions could be paired with either a lower or a higher SD in the same session. We observed no contextual effects on initial learning rates or on prediction error coding. This result implies that prediction errors adapt to SD rather than context.

The observed relationship between behavioral adaptation and task performance is in line with a previous study by our group (Diederen and Schultz, 2015). Diederen and Schultz (2015) observed that increases in prediction error scaling benefitted performance. However, observed over-scaling in this study resulted in performance decreases, resulting in a quadratic relationship between prediction error scaling and task performance. We did not observe such over-scaling in the present study and thus found a linear relationship between prediction error scaling and task performance. In addition, the current study used a related, but different, measure for prediction error scaling (i.e., behavioral adaptation) to facilitate similarity to the measure for adaptive coding.

We observed a correlation between trial-by-trial learning rates and BOLD responses in the occipital cortex and cerebellum, which has been reported previously (Krugel et al., 2009, McGuire et al., 2014, Payzan-LeNestour et al., 2013). This result suggests that the effect of trial number on learning is regulated via the learning rate. The occipital cortex is involved in the direction of visual attention toward task stimuli (Carter et al., 1995, Corbetta, 1998, Hahn et al., 2006). It has therefore been hypothesized that increases in learning rate reflect increased visual attention toward reward stimuli (Payzan-LeNestour et al., 2013). In our task, earlier rewards are more informative than later ones, as reflected in higher learning rates, which would suggest increased visual attention to earlier outcomes. Alternatively, occipital activation may be related to the yellow bars that indicated the magnitude of the prediction error on each trial. Although occipital responses did not vary with prediction errors, neurons in the occipital cortex may have visually adapted to the yellow bars across trials leading to decreases in visual responses, in parallel with decreases in learning rate. Interestingly, a correlation between learning rate magnitude and cerebellar activity was only observed previously when changes in learning rate depended on reward magnitude, but not when the learning rate depended on belief uncertainty and outcome volatility (McGuire et al., 2014). This finding is in line with the current study as our participants presumably updated their learning rates as a function of the RPE.

It has to be noted that the spatial resolution used in this study limited our ability to localize BOLD signal changes in the SN/VTA and the ventral striatum. Although we limited anatomical uncertainty through the use of functional ROIs that were constrained by anatomical masks, future studies are required that include higher spatial resolution and anatomical specificity. Another limitation pertains to the possibility that participants scaled their prediction errors because they were informed that their pay-off was scaled by SD in the control trials. Thus, our results show that prediction errors can scale with SD.

## Experimental Procedures

### Experimental Task

Twenty-seven participants predicted the magnitude of upcoming reward as closely as possible from the past reward history. Reward (£s) were drawn from one of six pseudo-Gaussian distributions with a SD of £5, £10, or £15 and an EV (mean) of £35 or £65 (see Supplemental Experimental Procedures).

Trials started with a fixation cross presented on a computer monitor in front of the participants (Figure 1A). After 2,100–4,200 ms of fixation cross presentation, a visual cue signaled (500 ms) the SD of the reward distribution from which the upcoming reward would be drawn. Cues were gray vertical rectangles intersected by two horizontal green bars. The vertical distance between the green bars signaled whether rewards were drawn from a distribution with a small, medium, or large SD (Figure 1A, inset). Distance was proportional to SD but did not correspond to the actual SD. Thus, it indicated whether rewards were drawn from a distribution with a small, medium, or large level of variability without informing participants about the actual SD. These explicit cues facilitated instantaneous adaptation to reward variability. Bar cues contained no explicit information about the EV of the reward. Following the cue, participants moved a horizontal “prediction” bar on a vertical scale that indicated the range of possible predictions (£0–£100) using a trackball mouse. Prediction value (in £) was displayed on both sides of the prediction bar and increased or decreased as participants moved the bar. Participants indicated their prediction by a mouse click (within 3,500 ms). The prediction bar appeared at a random position on the vertical scale at the start of each trial to decorrelate prediction magnitude from scrolling distance. After a variable delay (2,100–5,250 ms uniform distribution), which allowed BOLD responses for prediction and reward to be differentiated, the display showed the magnitude of the actual drawn reward as a green line and figure (corresponding to the monetary value of the reward) on the same scale, as well as the RPE on that trial (a yellow bar spanning the distance between the lines for the predicted and the received reward). Failure to make a timely prediction resulted in omission of the reward. Initial inspection of RPE data revealed that these errors increased with SD, indicating that the experimental manipulation was successful (Figure 1B). Participants were instructed on the experiment with the aid of a standardized tutorial, presented using MATLAB, which fully informed them about the structure of the task (see Supplemental Experimental Procedures).

To investigate whether task performance was related to individual working memory capacity, we administered the Wechsler reverse Digit Span task (Wechsler, 1958). Scores on this measure reflect the longest list of numbers that a person can correctly repeat in reverse order immediately after presentation. All stimulus presentation, data acquisition and behavioral analyzes were programmed using MATLAB (MathWorks) and Cogent 2000 (http://www.vislab.ucl.ac.uk/cogent_2000.php).

### Incentive Compatibility

We pseudo-randomly interspersed unannounced control trials (20%) into the main task to ensure that participants revealed their true predictions. Pay-off in control trials depended on performance (|prediction − EV|; Supplemental Experimental Procedures). In the main trials (80%), the pay-off was a fraction (10%) of the reward drawn by the computer (e.g., £5 if a participant received £50). This design motivated the participants to consider the drawn numbers actual reward. At the end of the experiment, the gains from 1 control and 1 main trial were selected randomly and paid out to the participants who had been informed about this pay-off procedure.

### Computational Models

To infer participants’ strategy on the task, we fitted a number of computational models to participants’ prediction sequences. We consider the case in which participants’ predictions (*y*) are assumed to result from a recursive generative process:

(Equation 1) $$y_{n}=y_{n-1}+k_{n}\delta_{n}$$

where *k*_*n*_ denotes the learning rate (also termed Kalman gain) and *δ*_*n*_ denotes the RPE on trial *n.* Thus, all models contain an error-driven update. The different models, which we fit to the participants’ prediction sequences, varied in the calculation of the learning rate, which indicates the degree to which the RPE on trial *n* is used to update the prediction on trial *n +* 1.

### Bayesian Mean-Tracking

Optimal performance on this task is achieved through accurate estimation of the EV of reward distributions. Optimal estimation of the EV can be derived using Bayes’ rule, a specific form of statistical reasoning (see Supplemental Experimental Material). Thus, a Bayesian mean-tracker constituted the null model for our task. The learning rate for an optimal mean-tracker in these experiments is

(Equation 2) $$k_{n}=\frac{\sigma_{n-1}^{2}}{\sigma_{n-1}^{2}+\sigma^{2}},$$

where *σ*^2^ is the variance of the reward and $\sigma_{n-1}^{2}$ is the variance of the prior. For the Bayesian mean tracker, the posterior variance decreases on every trial leading to asymptotic update of the posterior mean. Thus, predictions would not change much after many observations.

### RW

As Bayesian mean-tracking is computationally demanding, it is conceivable that participants use computationally more tractable approaches such as model-free reinforcement learning. We first consider the most basic reinforcement-learning rule:

(Equation 3) $$k_{n}=\alpha$$

in which the gain is the constant RW (Rescorla and Wagner, 1972) learning rate α. Using this model, participants update their predictions as a constant fraction of the RPE.

#### PH

When rewards are drawn from a Gaussian process, constant (RW) learning rates interfere with the acquisition of stable predictions. In addition, the use of constant learning rates would not be compatible with the instruction given to the participants that the reward are drawn from an approximate Gaussian distribution with a constant mean. Thus, it seems reasonable to consider a middle ground between Bayesian updating and constant learning such as the PH (Pearce and Hall, 1980) associability:

(Equation 4) $$k_{n}=\gamma C\left|\delta_{n-1}\right|+\left(1-\gamma \right)k_{n-1},$$

where |*δ*| denotes the absolute RPE and *C* is an arbitrary scaling coefficient. The recursive process is initialized with the initial learning rate *k*_0_ = *α*. In this case, the learning rate depends on the absolute RPE on previous trials, the learning rate on previous trials, and the decay constant γ.

### Adaptive PH

To account for the potential effect of SD in the PH model, we scaled the prediction error relative to log(SD) of the reward distributions.

$$y_{n}=y_{n-1}+k_{n}\delta_{n}/\omega$$

$$k_{n}=\gamma C\left|\delta_{n-1}\right|/\omega +\left(1-\gamma \right)k_{n-1}$$

(Equation 5) ω=(1−ν)+νlog(SD)/D,

Since scaling compresses the operational range of the learning rate to update predictions, we added an arbitrary scaling coefficient *D* to ensure scaling relative to, but with a quantity smaller than log(SD). In addition, as we previously showed individual variation in the degree of prediction error scaling, we estimated the extent of prediction error scaling (0 ≤ *v* ≤ 1) per participant (Diederen and Schultz, 2015).

### Model Fitting and Comparison

For each model, we fit the free parameters Φ to the subjective predictions *Y* by maximizing the likelihood $p\left(Y|\Phi \right)=\prod_{m}^{M}p\left(y_{m}|\Phi \right)$, where $p\left(y_{m}|\Phi \right)=N\left(\mu_{m},{\hat{\sigma }}^{2}\right)$ and $Y=\left[y_{1}\;y_{2}\;..\;y_{M}\right]$ are the subjective predictions. We used a combination of nonlinear optimization algorithms implemented in MATLAB to estimate the free parameters to each participant’s full dataset over the trials of all conditions. Formal model comparisons were conducted using Akaike Information Criterion (AIC) and Bayesian Information Criterion (BIC) values that take into account the difference in the numbers of free parameters between models (see Supplemental Experimental Material).

### fMRI

fMRI data were obtained at the Wolfson Brain Imaging Center, Cambridge, using a Siemens Trio 3T MRI scanner (see Supplemental Experimental Procedures).

### Adaptive Prediction Error Responses

Our first fMRI analysis investigated whether BOLD responses would adapt to the variability of RPEs. If the brain’s limited coding capacity adapts to variability in RPEs, BOLD responses should increase less for a given increase in RPE. This mechanism would result in shallower slopes for coding RPEs with larger SDs (Figure 3A, left). Without such adaptation, regression slopes would be similar for the different SDs (Figure 3B, right). Thus, the brain’s sensitivity to small changes in RPEs would be lower for distributions with larger SDs.

To test for adaptive RPE coding, we created a single regression model for each participant. We modeled cue onset, prediction onset and reward onset as single impulse responses. Events were created separately for each SD. Furthermore, reward onset events were separately modeled for trials with a positive RPE and trials with a negative RPE as previous studies reported stronger responses for negative RPEs in the human SN/VTA complex and striatum (D’Ardenne et al., 2008, Liu et al., 2011). We parametrically modulated reward onset events with trial-wise (1) reward outcome value and (2) RPEs. The RPE parametric modulator was orthogonalized with respect to the outcome value parametric modulator to ensure that this parametric modulator indicated BOLD responses varying with RPEs, independently of reward magnitude. The analyses included the main trials (80%) and the control trials (20%) as the participants indicated that they treated all trails in the same fashion. Specifically, the participants aimed to predict upcoming reward as well as possible from the past reward history, and they favored higher reward at the outcome phase. In addition, preliminary analyses including only the main trials revealed comparable results to the analyses including all task trials. To account for a maximal number of variables influencing brain activity, we included covariates. Covariates consisted of error trials (trials in which participants failed to indicate their prediction within 3,500 ms) and the prediction time (time between initial appearance of the scale and the moment participants stated their prediction) in non-error trials. These epochs and all events were convolved with the standardized hemodynamic response function from SPM8 to introduce typical delays of fMRI responses. Finally, we modeled movement artifacts by including the realignment parameters as regressors of no interest. All regressors were fitted to the data using general linear model estimation.

After model estimation, linear contrasts of regression coefficients of interest were computed at the individual level and then entered in second level random effects repeated-measures ANOVAs to test for group effects. We carried out the following contrast: Main effect RPE adaptation (SD5 > SD10 > SD15); this contrast revealed regions where BOLD responses to positive and negative RPEs varied more strongly with RPEs when the SD was smaller, independent of outcome value.

### Normalized Coding of Prediction Errors

The above analysis aimed to investigate whether BOLD responses adapted to the variability of RPEs. If so, this would render it likely that these errors are encoded in a normalized fashion (i.e., as a function of SD). As before, we modeled cue onset, prediction onset, and reward onset as events, and reward onsets separately for positive and negative RPEs. All events were collapsed over the different SDs. Reward onset regressors were parametrically modulated with (1) outcome value, (2) non-normalized RPEs, and (3) normalized RPEs (RPEs/SD). As we were interested in variance uniquely explained by each of these parametric modulators, we removed the serial Gram-Smidt orthogonalization procedure from the analysis. This procedure ensured that shared variance between outcome value and normalized and non-normalized RPEs would be excluded from the analysis, rather than being attributed to one of the parametric modulators. This is a highly conservative procedure for partly correlated regressors, as the shared variance goes in the residuals thus limiting the statistical quality of the parametric modulator. To facilitate comparison of normalized and non-normalized regressors, parametric modulators were *Z* scored prior to model estimation. *Z* scores were calculated per subject, across all SD conditions. As behavioral adaptation involves learning rate decay in addition to RPE scaling, we ran an additional model. Here, the second parametric modulator consisted of RPEs that were multiplied with dynamic trial-wise learning rates estimated across different SD conditions in addition to RPE scaling. Error trials and prediction time were included as covariates, and the realignment parameters were included as regressors of no interest.

### Context Dependency

Each session included two conditions differing in SD and alternating in short blocks. Thus, RPE coding slopes could adapt to the relative SDs (high or low) of each condition within a session. As SD 10 conditions could be paired with either a lower or higher SD condition in a session, we investigated this hypothesis by comparing RPE regression slopes for the two SD 10 conditions.

### Learning Rate Coding

Whereas we hypothesized that the weight attributed to RPEs as a function of SD would be reflected in the coding of normalized RPEs, the weight attributed to RPEs might alternatively be reflected in the coding of SD-dependent dynamic learning rates. To investigate this alternative explanation, we repeated the first fMRI model and used the estimated PH dynamic learning rate rather than RPE as the second parametric modulator.

### Timescale Adaptive Coding

As the adaptive process conceivably requires time, we investigated the timescale for SD adaptation. With this aim, we modified our first model so that reward onsets were modeled separately for early trials (1–7), middle trials (8–14), and late trials (15–21). As responses for each SD were averaged for the two EVs, RPE responses for early, intermediate, and late learning were estimated for 14 trials. Here, reward onset events were combined for positive and negative predictions errors to ensure a sufficient number of observations for each condition. In addition, we included no parametric modulators besides RPEs as reliable estimation of regression slopes for partially correlated parameters is unfeasible with a small number of observations. For each participant and each timescale, we estimated SD-specific RPE coding slopes in the a-priori-defined SN/VTA complex and ventral striatum. Extracted parameter estimates were averaged over the left and right (1) SN/VTA complex and (2) ventral striatum.

### Thresholding

Adaptive coding effects are likely to be subtle as previous fMRI studies only reported results that were uncorrected for multiple comparisons (Bunzeck et al., 2010, Park et al., 2012). Thus, we performed analyses in an a-priori-defined ROI that comprised the midbrain SN/VTA complex and ventral striatum as well as on whole-brain level. First, we traced the SN/VTA complex on a normalized high-resolution magnetic transfer image acquired using the same MRI scanner as the functional MR images (Gruber et al., 2014). In addition, the ventral striatal ROI was traced on the average T1 scan of our participants following the definition of the ventral striatum by Laruelle et al. (Martinez et al., 2003). To increase sensitivity to identify effects within these ROIs, we inclusively masked the anatomical ROI with clusters of significant RPE related activation reported in a recent meta-analysis (Gruber et al., 2014; meta-analysis data provided by Garrison et al., 2013) (see Figure S1 for an illustration of our ROI). The SN/VTA complex and ventral striatum were combined into one ROI to ensure that corrections for multiple comparisons were conducted across all voxels in both areas. We also constructed a control ROI of the same dimensions as the a-priori-defined ROI to ensure that any significant results in the a priori ROI did not solely result from the more liberal multiple comparisons correction. The control ROI was centered at the cerebellum (i.e., −30/30, −76-40). For the a priori and control ROI, we considered activations significant at p < 0.05 family-wise error (FWE) corrected using a SVC. On whole-brain level we report results p < 0.05, FWE corrected at the cluster level as well as results p < 0.05 FWE corrected at the voxel-level.

### Adaptation to Reward Variability and Task Performance

We hypothesized that neural adaptation to SD would facilitate behavioral adaptation to SD and that the individual degree of adaptation would correlate with task performance. To investigate the hypothesized relationships, we first quantified the individual degree of behavioral and neural adaptation to the SD of reward. Behavioral adaptation would be reflected in the effect of SD on the estimated learning rates. Similarly, SD-dependent variation in RPE coding slopes would be indicative of neural adaptation. In line with previous findings of a non-linear relationship between SD and learning rates (Diederen and Schultz, 2015), we used the inverse of SD as predictor. Thus, we quantified in each participant whether SD^−1^ was a significant predictor of learning rates and RPE coding slopes: *β*_0_ + *β*_1_ SD^−1^. The R^2^ of these regression analyses reflect the individual degree of adaptation: higher R^2^ indicated that SD was a better predictor of learning rate and RPE slopes. Since the primary focus was the effect of SD on learning rates and RPE slopes, we dissociated the effect of SD from the effect of trial number on learning rates and RPE slopes in these analyses. Subsequently, we related the individual degree of behavioral and neural adaptation to task performance. Efficient learning requires individuals to rapidly acquire stable and accurate predictions in contexts with varying degrees of reward variability. Thus, we quantified task performance as the performance error (|prediction − EV|) averaged across all trials. Importantly, performance error reflects both prediction accuracy and stability. Specifically, large performance errors could result from unstable predictions indicating that learning had not yet been completed, as well as from stable predictions with low accuracy (i.e., distant from the EV). We calculated rank correlations (Spearman’s ρ) to establish the relationship between behavioral and neural adaptation and between adaptation and task performance, as this data was not normally distributed.

## Author Contributions

K.M.J.D., W.S., and P.C.F. designed the experiment; K.M.J.D. and T.S. collected data; K.M.J.D. and M.D.V. performed analyses; K.M.J.D., W.S., P.C.F., M.D.V., and T.S. wrote the paper.

## Figures and Tables

**Table 1:** Quality of the Generative Models Fitted to Behavioral Data Given as the Mean Difference (d) in Criterion Values (AIC and BIC) across Participants

Model	Bayes	RW	PH
**RW**			

*d*AIC	−2.8		
*d*BIC	−2.4		

**PH**			

*d*AIC	−7.4	−10.2	
*d*BIC	−7.9	−5.5	

**Adaptive PH**			

*d*AIC	−8.0	−10.5	−3.8
*d*BIC	−5.2	−4.8	−1.0

RW, Rescorla-Wagner; PH, Pearce-Hall.

Since SD is a key parameter of the Bayesian model, we fitted this model separately for each SD condition and compared the resulting fits to similarly obtained fits for the RW and the PH model. As the main difference between the PH models is the SD-dependent change in learning rate (implemented using a single scaling parameter), we used model fits across SD conditions to compare the adaptive PH model to the non-adaptive models. While model comparisons using AIC provided strong evidence in favor of the adaptive PH model, BIC only showed a marginal improvement of the adaptive PH model over the non-adaptive variant.

**Table 2:** Whole-Brain Adaptive Coding

Brain Area	Cluster Size	Max. Z Value	Cluster p Value	MNI Coordinates		
X	Y	Z
Supramarginal gyrus	452	5.05	0.000	−46	−8	2
Parahippocampal gyrus				−18	−40	−5
Superior temporal gyrus	182	4.94	0.000	54	−44	18
Supramarginal gyrus				58	−40	34
Middle frontal gyrus	76	4.72	0.002	−26	28	42
Parahippocampal gyrus	43	4.74	0.007	22	−44	−2
Thalamus				14	−28	−5
Middle temporal gyrus	37	4.56	0.011	50	−12	−14
Superior temporal gyrus				45	−12	−5

Cluster sizes, p values, z values, and locations of local maxima for brain regions, other than the SN/VTA complex and ventral striatum, showing adaptive coding of prediction errors to reward variability.

## Supplemental Information

Document S1. Figures S1–S3, Table S1, and Supplemental Experimental Procedures

Document S2. Article plus Supplemental Information
