# Supplementary material for: Adaptive Prediction Error Coding in the Human Midbrain and Striatum Facilitates Behavioral Adaptation and Learning Efficiency
Source: Neuron. 2016 Jun 1;90(5):1127–38. doi: 10.1016/j.neuron.2016.04.019 (PMC4893165; doi:10.1016/j.neuron.2016.04.019)
Supplement: Document S1. Figures S1–S3, Table S1, and Supplemental Experimental Procedures [file mmc1.pdf]

Neuron, Volume *90*

Supplemental Information

Adaptive Prediction Error Coding in the Human Midbrain and Striatum Facilitates Behavioral Adaptation and Learning Efficiency

Kelly M.J. Diederen, Tom Spencer, Martin D. Vestergaard, Paul C. Fletcher, and Wolfram Schultz

Supplemental Inventory

1. Supplemental Figures

Figure S1. Related to Experimental Procedures
Figure S2. Related to Figure 1
Figure S3. Related to Figure 1

2. Supplemental Tables

Table S1. Related to Experimental Procedures.

3. Supplemental Experimental Procedures

4. Supplemental References

Supplemental Figures

Figure S1. A priori defined region of interest (ROI).

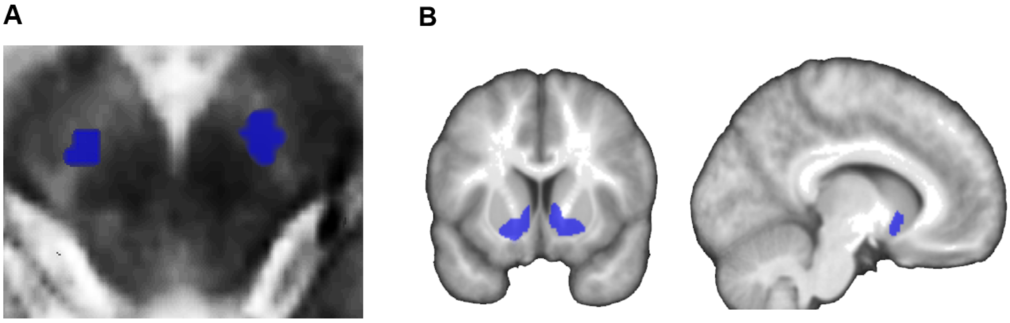

A. Midbrain (SN/VTA) ROI depicted in blue on a magnetic transfer imaging scan. The SN/VTA complex is visible as a light grey band. As adaptive coding effects are likely to be subtle, we constructed maximum sensitive ROIs by using a functional ROI that was restricted by anatomical boundaries in line with the procedure by Gruber et al. (2014). We traced the SN/VTA complex (light grey band) on a normalized magnetic transfer image acquired using the same MRI scanner as the functional MR images. Subsequently, we inclusively masked the anatomical ROI with clusters of significant prediction error related activation reported in a recent meta-analysis (data provided by Garrison et al., 2013). B. Ventral striatal ROI (blue). The ventral striatal ROI was traced on the average T1 scan of our participants following the definition of the ventral striatum by Laruelle et al. (Martinez et al., 2003). As with the SN/VTA ROI, we inclusively masked this anatomical ROI with prediction error related activation reported in a recent meta-analysis (data provided by Garrison et al., 2013).

Figure S2. Main model parameters fitted to participants' behavior for separate SD conditions

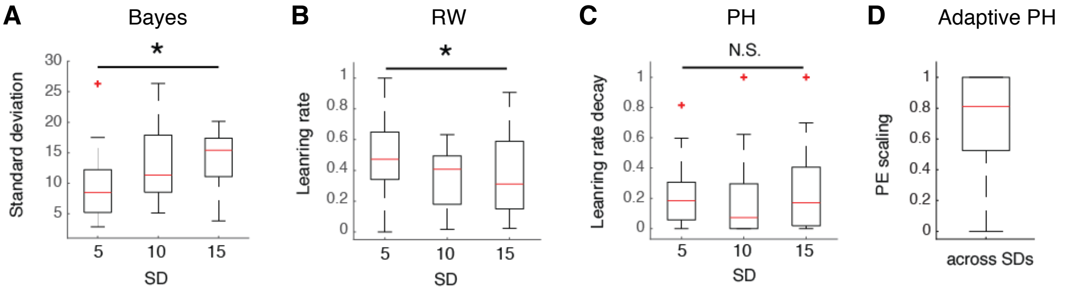

A. In the Bayesian model, the free parameter σ^2 indicated participants' estimates of the variance associated with each SD condition. Here we plot the standard deviation, i.e., the square root of the variance. Participants' estimates of the variance increased in parallel with actual increases in reward variance. B. Fitted Rescorla-Wagner (RW) constant learning rates decreased when SD increased, in line with behavioral adaptation and the (initial) learning rates estimated for the non-adaptive Pearce-Hall model (supplemental experimental material). C. The gradual decay in learning rate as described in the Pearce-Hall (PH) model did not vary between SD conditions, indicating that the effect of trial number did not interact with SD. D. The free parameter ν indicates the extent to which participants scaled their prediction errors in the adaptive PH model (supplemental experimental procedures). A parameter value of 0 indicates absence of prediction error scaling, whereas a value of 1 indicates that participants divide their value by the log(SD) of reward distributions. * denotes significant; N.S., not significant. SD, standard deviation; RW, Rescorla-Wagner; PH, Pearce-Hall; PE, prediction error. Boxplots indicate the minimum and maximum parameter estimates excluding outliers, the lower and upper quartile and the median (red line).

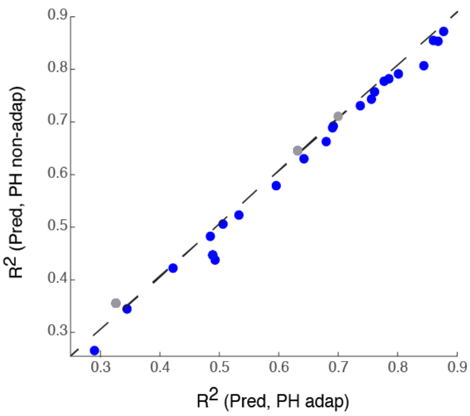

Figure S3. R^2 values from linear regressions where modeled predictions from the non-adaptive (Eq. 4) and adaptive (Eq. 5) Pearce-Hall models were the independent variables and participants' predictions were the dependent variable. Although the differences between the R^2 for the two models are subtle, most participants' predictions were better explained by the adaptive Pearce-Hall model. Indeed, predictions generated by the adaptive PH model were a significantly better predictor of participants' predictions than the non-adaptive PH model ($T(26) = 2.56$, $p = 0.0083$). Blue/ grey dots represent participants whose behavior was best predicted by the adaptive/ non-adaptive Pearce-Hall model.

Supplemental Tables

Table S1: Description of free parameters fitted for each model per SD condition.

Model	#Φ	Parameters
Bayes	2	σ_0^2, σ^2
RW	1	α
PH	2	α, γ
Adaptive PH	3	α, γ, υ

See Fig. S2 for the main parameter estimates per SD condition.

Supplemental Experimental Procedures

Participants. We recruited twenty-seven healthy volunteers (11 male; 16 female) through local advertisements. Participants were between 18 and 41 (mean 24.49, s.e.m. 1.06) years of age; they were fluent English speakers and did not have a history of a neurological or psychiatric illness or drug abuse. This study was approved by the Local Research Ethics Committee of the Cambridgeshire Health Authority. After description of the study to the Participants, written informed consent was obtained.

Reward distributions. All reward distributions contained 21 rewards which were drawn without replacement, thus ensuring that each participant received the same rewards. Each participant completed three task sessions of 10 min each during fMRI data acquisition. Every session used two reward distributions drawn pseudo randomly from the six distributions, resulting in 42 trials per session (i.e., 21 trials per distribution; 2 distributions per session). The order of rewards within a distribution was counterbalanced over participants. Importantly, both the EV and the SD of the two distributions within a session were different, and each distribution occurred only once per participant. Distributions were presented in short blocks of 4-6 trials. There were six possible pairs of distributions, of which each participant saw three pairs (i.e., 1 pair per session). Fourteen participants were presented with the first combination of pairs (SD5 EV35 and SD10 EV65, SD10 EV35 and SD15 EV65, SD15 EV35 and SD5 EV65). The remaining thirteen participants performed the second combination (SD 5 EV35 and SD15 EV65, SD10 EV35 and SD5 EV65, SD15 EV35 and SD10 EV65). The order of rewards within a condition was pseudo-randomized. First, we randomized the rewards within a condition using Matlab. Subsequently, we ensured that outliers did not occur in succeeding trials. All distributions had zero skewness, no tails and non-significant deviation from normality (Shapiro-Wilk; p = 0.54, 0.89 and 0.92 for SD's of £5, £10 and £15). However, they were slightly less 'peaked' than a true Gaussian distribution as indicated by a kurtosis of 2.6 (SD 5), 2.6 (SD 10) and 2.57 (SD 15).

Instructions. We indicated to the participants that rewards were drawn from 'pots' (i.e., distributions) with a small, medium or large degree of variability as indicated by the bar cues. Furthermore, we informed participants that each of the three task sessions required them to alternatingly predict from one of two 'pots' (distributions) resulting in a total of six different pots (small variability N=2; medium variability N=2 and large variability N=2). We explicitly stated that all changes in condition would be signalled using the bar cues. Participants were only ignorant about the exact parameter values (i.e., the EVs and SDs used as well as the frequency of alternation between the two distributions within a session). Debriefing after the experiment revealed that participants believed that each of the six distributions had a different EV. We informed the participants that the goal of the experiment was to predict the next reward as closely as possible from the past reward history. As the imposed variability would render it unlikely for participants to achieve full accuracy predicting upcoming rewards, we instructed participants to minimize their total error over all trials.

Practice sessions. To familiarize participants with a trackball mouse, participants completed a short motor task prior to the main task. In each trial (total of 90 trials) participants were required to scroll to a specific number on the scale, indicated in green on top of the scale. In addition, participants completed two behavioral training sessions prior to the fMRI experiment using rewards drawn from distributions with a different SD (i.e., £7 and £14) and EV (i.e., £30 and £60). We proceeded to the fMRI experiment if participants were fully aware of all task contingencies except for the exact SDs and EVs used.

Control trials. We pseudo randomly interspersed, unannounced control trials (20% of all trials) into the main task to ensure that participants revealed their true reward predictions. Pay-off in these control trials depended on performance (|*prediction - EV*|). Prediction error magnitude within one or two SDs of the EV resulted in a pay-off of £7.50 and £5.00, respectively. All other predictions led to a pay-off of £2.50. As in the main trials, the monitor displayed the reward drawn by the computer after the participant had indicated the prediction. However, the reward was shown in red to signal that in this trial prize money/ pay-off depended on participants' performance. Thus, importantly, there was no indication about the control trial at the time the participants stated their predictions, encouraging participants to optimize their performance on all trials.

Reward process. The reward x on every trial is drawn from a distribution with a Gaussian prior $x \sim \mathcal{N}(\mu, \sigma^2)$. In the main text, we refer to the expected value ($\mathrm{EV} = \mu$) and to the standard

deviation (SD $= \sigma$) of the reward. On trial n, participants predict to receive reward y_n and they observe the prediction error $\delta_n = x_n - y_n$.

Models. We consider cases, in which the participants' predictions are assumed to result from a recursive generative process, $y_n = y_{n-1} + k_n \delta_n$, where k_n denotes the Kalman gain (i.e., learning rate).

1. Bayesian mean tracker. Optimal performance on this task is achieved through accurate estimation of the EV of the reward. An optimal estimator of the Gaussian prior μ is derived from Bayes' rule. The conjugate prior is $\mu \sim \mathcal{N}(\mu_0, \sigma_0^2)$, and given an observation $X = [x_1\ x_2 \ldots x_N]$, the log-likelihood of the posterior $\mu \sim \mathcal{N}(\mu_N, \sigma_N^2)$, is given by:

$$\log[p(\mu|X)] = -\frac{1}{2\sigma_N^2}(\mu - \mu_N)^2 + K_1 = -\frac{1}{2\sigma_N^2}\mu^2 + \frac{\mu_N}{\sigma_N^2}\mu + K_2$$

From Bayes' rule, we have $p(\mu|X) \propto p(X|\mu, \sigma^2)p(\mu|\mu_0, \sigma_0^2)$, and so:

$$\log[p(\mu|X)] = -\frac{1}{2\sigma^2}\sum\nolimits_n^N (x_n - \mu)^2 - \frac{1}{2\sigma_0^2}(\mu - \mu_0)^2 + K_3$$
$$= -\frac{1}{2}\left[\frac{1}{\sigma_0^2} + \frac{N}{\sigma^2}\right]\mu^2 + \left[\frac{\mu_0}{\sigma_0^2} + \frac{\sum_n^N x_n}{\sigma^2}\right]\mu + K_4$$

where K_i are constant terms. Thus, since:

$$\frac{1}{2\sigma_N^2} = \frac{1}{2}\left[\frac{1}{\sigma_0^2} + \frac{N}{\sigma^2}\right]$$

the posterior variance is:

$$\sigma_N^2 = \frac{\sigma^2\sigma_0^2}{N\sigma_0^2 + \sigma^2}$$

Similarly, since:

$$\frac{\mu_N}{\sigma_N^2} = \frac{\mu_0}{\sigma_0^2} + \frac{\sum_n^N x_n}{\sigma^2}$$

the posterior mean is:

$$\mu_N = \frac{\sigma^2}{N\sigma_0^2 + \sigma^2}\mu_0 + \frac{N\sigma_0^2}{N\sigma_0^2 + \sigma^2}\bar{X}$$

where $N\bar{X} = \sum_n^N x_n$.

We consider the case, in which participants update the prior after each observation ($N = 1$). This seems reasonable since a subjective prediction is required in response to every prediction error after each reward.

$$\mu_n = \frac{\sigma^2}{\sigma_{n-1}^2 + \sigma^2}\mu_{n-1} + \frac{\sigma_{n-1}^2}{\sigma_{n-1}^2 + \sigma^2}x_n = \mu_{n-1} + \frac{\sigma_{n-1}^2}{\sigma_{n-1}^2 + \sigma^2}(x_n - \mu_{n-1})$$

Therefore, the Kalman gain (i.e., learning rate) for an optimal mean tracker in these experiments is:

$$k_n = \frac{\sigma_{n-1}^2}{\sigma_{n-1}^2 + \sigma^2}$$

The posterior prediction is $y_n \sim \mathcal{N}(\mu_n, \hat{\sigma}_n^2)$, where $\hat{\sigma}_n^2 = \sigma_n^2 + (1 - k_n)\sigma_{n-1}^2$.

As participants may have differed in their estimates of reward variability, we estimated the most likely value of σ^2 used by each individual participant. Moreover, since we only used two different EVs in the main task, participants had the opportunity to build strong priors between sessions. However, the participants' posterior means (i.e., final predictions) in the first session did not show a significant positive correlation with the first predictions in the second session (all p > 0.1). Similarly, the final predictions in the second session did not show a significant positive correlation with the initial predictions in the third session (all p > 0.1). Therefore, we did not include structural priors in the Bayesian model.

2. Rescorla-Wagner learning rule (RW; Rescorla and Wagner 1972). The RW model is one of the most influential theories of associative learning in human and particularly animal learning theory. In this simple associative learning model, individuals are assumed to use a constant learning rate that controls how much an observed prediction error will influence new predictions:

$$k_n = \alpha$$

In this case, predictions are assumed to be generated by constant learning.

3. Pearce-Hall (PH; Pearce and Hall, 1980). Although RW may facilitate stable predictions when reward magnitude is constant, a fixed learning rate will result in varying predictions when rewards fluctuate, i.e., participants persistently 'chase the prediction error'. Stable predictions may, however, be achieved through the use of a decaying learning rate as described in the PH associability model:

$$k_n = \gamma C|\delta_{n-1}| + (1-\gamma)k_{n-1}$$

where $|\delta|$ denotes absolute prediction error and C is an arbitrary scaling coefficient. We combine the PH associability (learning rate) with the recursive generative process described above in line with the procedure suggested by Li et al (2011). The recursive process is initialized with the initial learning rate $k_0 = \alpha$. In this case, predictions are assumed to be generated under decaying learning rate with the decay constant γ. Importantly, learning rates depend on the absolute prediction error and the learning rate on the previous trial as well as on the decay constant γ. A critical feature of this model is that it allows for the combination of high initial learning rate and exponential decay enabling substantial initial updating as well as asymptotically stable later predictions. Moreover, while SD may influence the initial learning rate as well as the decay constant, we have previously shown that the effect of SD was primarily on the initial learning rate (Diederen and Schultz, 2015).

4. Adaptive Pearce-Hall (Diederen and Schultz, 2015). To account for the potential effect of SD in the PH model, we scaled the prediction error relative to log(SD) of the reward distributions. Note that an improved fit by this model indicates that non-scaled PH learning rates vary with SD. The rationale for scaling the prediction error rather than the learning rate was that previous non-human primate electrophysiology studies showed encoding of normalized PEs, not learning rates (Tobler et al., 2005). Since scaling compresses the operational range of the learning rate to update predictions, we added an arbitrary scaling coefficient D to ensure scaling relative to, but with a quantity smaller than log(SD). In addition, as we previously showed individual variation in the degree of prediction error scaling, we estimated the extent of prediction error scaling ($0 \leq \nu \leq 1$) per participant (Diederen and Schultz, 2015):

$$y_n = y_{n-1} + k_n\delta_n/\omega$$
$$k_n = \gamma C|\delta_{n-1}|/\omega + (1-\gamma)k_{n-1}$$
$$\omega = (1-\nu) + \nu\log(\mathrm{SD})/D$$

Here, ν indicates the extent of prediction error scaling. The form of this update rule ensured that the model could return both the absence of scaling (ν = 0) as well as scaling by the log(SD) (ν = 1).

Model fitting. For each model, we fit the free parameters Φ to the subjective predictions Y by maximizing the likelihood $p(Y|\Phi) = \prod_m^M p(y_m|\Phi)$, where $p(y_m|\Phi) = \mathcal{N}(y_m, \hat{\sigma}^2)$ and $Y = [y_1\, y_2\, ..\, y_M]$ is the subjective predictions. We used a combination of nonlinear optimization algorithms implemented in MATLAB to estimate the free parameters to each participant's full data set over the trials of all conditions. Since SD is a key parameter of the Bayesian model, we fit this model separately

for each SD condition and compared the resulting fits to similarly obtained fits for the RW and the PH model. In addition, as the main difference between the PH models is the SD-dependent change in learning rate (implemented using a single scaling parameter), we used model fits across SD conditions to compare the adaptive PH model to the non-adaptive models.

Functional MRI. FMRI data were obtained at the Wolfson Brain Imaging Center, Cambridge, using a Siemens Trio 3T MRI scanner. We acquired 240 multiecho gradient-echo echo planar T_2^*-weighted images depicting blood oxygenation level-dependent (BOLD) contrast for each session of the task (Poser et al., 2006). Imaging at multiple echo times has the potential to increase sensitivity in brain regions that are typically subject to strong image distortions (Poser et al., 2006). Each participant completed 3 task sessions, resulting in 720 volumes per participant. We used the following parameters for obtaining BOLD images: 30 axial slices (3.78 mm slice thickness), repetition time (TR) 2100 ms, echo times (TEs): 12/ 27.91/43.82/ 59.73 ms, flip angle 82°, field of view (FOV) 14.4x14.4 cm, matrix 64x64, in-plane resolution 3.75x3.75 mm. This resolution facilitated the detection of BOLD responses on whole-brain level. Whole brain coverage was of particular importance to investigate the alternative hypothesis that behavioral adaptation to reward variability is reflected in the coding of SD-dependent learning rates as learning rates are coded in frontal and occipital areas (Krugel et al. 2009; Payzan-LeNestour et al. 2013; Vilares et al. 2012). To improve localization of the functional data a high resolution anatomical scan was acquired during the same scan session (T_1; MPRAGE; TR/TE 2.98/2300 ms, 1x1 voxels, slice thickness 1 mm, flip angle 9°, FOV 24x25.6 mm, 176 slices).

Statistical parametric mapping (SPM8; Wellcome Department of Cognitive Neurology, London, UK) and MATLAB (MathWorks, Natick, MA) served to analyze and preprocess functional MRI data. Preprocessing included within-subject image realignment, voxelwise weighted echo combination (summation based on local T_2^* measurements) (Poser et al., 2006), coregistration of functional images with the T_1-weighted anatomical scan, spatial normalization to the Montreal Neurological Institute (MNI) template as present in SPM8 (Ashburner and Friston, 2005) and spatial smoothing using an 8mm full width at half maximum Gaussian kernel. To increase anatomic specificity, we repeated our preprocessing using a 6 mm smoothing kernel. The time-series in each session were high-pass filtered (1/180Hz) and serial autocorrelations were estimated using an AR(1) model.

Supplemental References

Ashburner J, Friston KJ (2005) Unified segmentation. NeuroImage 26:839-851

Poser, B.A., Versluis, M.J., Hoogduin, J.M., and Norris, D.G. (2006). BOLD contrast sensitivity enhancement and artifact reduction with multiecho EPI: Parallel-acquired inhomogeneity-desensitized fMRI. Magnetic Resonance in Medicine 55, 1227-1235.

Vilares, I., Howard, J.D., Fernandes, H.L., Gottfried, J.A., and Kording, K.P. (2012). Differential representations of prior and likelihood uncertainty in the human brain. Current biology : CB 22, 1641-1648.
